# Proposal for a Standard Protocol to Assess the Rheological Behavior of Thickening Products for Oropharyngeal Dysphagia

**DOI:** 10.3390/nu14235028

**Published:** 2022-11-25

**Authors:** Mireia Bolivar-Prados, Noemí Tomsen, Yuki Hayakawa, Satomi Kawakami, Kazuhiro Miyaji, Jun Kayashita, Pere Clavé

**Affiliations:** 1 Gastrointestinal Physiology Laboratory, Hospital de Mataró, Universitat Autònoma de Barcelona, 08304 Mataró, Spain; 2 Centro de Investigación Biomédica en Red de Enfermedades Hepáticas y Digestivas (Ciberehd), 28029 Madrid, Spain; 3 Health Care and Nutritional Science Institute, Morinaga Milk Industry Co., Ltd., Zama-City 252-8583, Japan; 4 Department of Health Sciences, Faculty of Human Culture and Science, Prefectural University of Hiroshima, Hiroshima 734-8558, Japan

**Keywords:** rheology, viscosity, shear rate, amylase, dysphagia

## Abstract

Increasing shear viscosity (ShV) in thickening products (TP) is a valid therapeutic strategy for oropharyngeal dysphagia (OD). However, salivary amylase in the oral phase and shear rate in the pharyngeal phase of swallowing can change the viscosity of TPs when swallowed. This study aims to design and validate a rheological protocol to reproduce the oral and pharyngeal factors that affect the therapeutic effect of TPs and report the viscosity measurements in a standardized scientific and precise manner. We measured (a) the variability of the ShV measurements across several laboratories; (b) the in vitro and ex vivo properties of TPs and (c) the impact of the X-ray contrast Omnipaque, temperature and resting time on the rheological properties of TPs. A common protocol was applied in four international laboratories to assess five ShV values (100, 200, 400, 800 and 1600 mPa·s) for the xanthan-gum TP Tsururinko Quickly (TQ). The protocol included the dose (g/100 mL water), stirring procedure and standing time before measurement. Each value was characterized at the shear rate of 50 and 300 s^−1^ pre- and post-oral incubation in eight volunteers. The effect of temperature, standing time and Omnipaque was assessed. The main results of the study were: (a) The mean intra-laboratory variability on the ShV at all levels was very low: 0.85%. The mean inter-laboratory variability was higher: 9.3%; (b) The shear thinning of TQ at 300 s^−1^ was 75–80%. Increasing the temperature or standing time did not affect the ShV, and oral amylase caused a small decrease; (c) Omnipaque slightly decreased the dose of TP and hardly affected the amylase resistance or shear thinning. This study showed that different laboratories can obtain very accurate and similar ShV measurements using this protocol which uses scientific, universal SI units (mPa·s). Our protocol accurately reproduces oral and pharyngeal factors affecting the therapeutic effect of TPs. The addition of X-ray contrast did not produce significant changes.

## 1. Introduction

A thickening product (TP) is a hydrocolloid used to increase the viscosity of a fluid, water or alimentary fluid in which it is mixed. These products are considered a valid strategy to treat oropharyngeal dysphagia (OD) [1,2,3,4,5,6] and are included under the category of Food for Special Medical Purposes (FSMPs) [7]. FSMPs are alimentary products used to manage the dietary conditions of patients suffering from a specific pathology and needing to be under medical supervision. The main hydrocolloids used for OD are modified starch (MS) and xanthan gum (XG) but other hydrocolloids such as guar and tara gum are also widely used in this specific field. MS is produced by chemically modified hydrocolloids, and XG’s origin is microbial fermentation [2]. The source and disposition of the molecules of each TP confer different characteristics [2,8]: the high number of O-glycosidic bonds and their external disposition in MS TPs makes them highly affected by salivary amylase while the tridimensional structure of XG TPs protects the oral enzymes and gives higher stability over time. In clinical practice, MS TPs are progressively being replaced by XG TPs because of their great resistance to oral amylase and better palatability and stability [9,10]. 

Increasing shear viscosity has proved to increase the prevalence of safe swallows in several phenotypes of patients with OD: older [6], post-stroke [5,6], head and neck cancer and neurodegenerative diseases [6]. However, the therapeutic effect of TPs depends on several factors such as the composition of the selected TP, the preparation method, the amount of TP, the thickness and the rheological parameters such as salivary amylase in the oral phase and shear thinning in the pharyngeal phase [11]. Shear viscosity has been identified as the main parameter linked to the therapeutic effect of TP in patients suffering from OD [5,6] and can be measured using the International System of Units in Pa·s [12]. In previous studies using XG TPs, we found a strong viscosity-dependent therapeutic effect on the safety of swallow at a range between 250–1000 mPa·s [6] and 250–800 mPa·s [5], depending on the TP. To measure the shear viscosity, the TP is submitted to different shear flows, and the apparent viscosity at each point is recorded in order to determine the viscosity values when the bolus is submitted to the shear forces when swallowing. TPs have non-Newtonian pseudoplastic behavior, which means that shear viscosity (ShV) decreases with the increment of shear forces [2,13,14] and needs to be tested at several shear rates to understand its behavior [15]. The shear rate spectrum for the whole swallowing process in humans is estimated to range between 1 to 1000 s^−1^ [15], and two major landmarks have been proposed to replicate the swallowing process in patients with OD. In the oral cavity, 50 s^−1^ has been recommended by the National Dysphagia Diet [16]. The second landmark to be considered is in the pharynx (specifically in the mesopharynx) where the head of the bolus reaches the laryngeal vestibule and can be aspirated. At this specific anatomic point, the shear rate has been determined at 262 s^−1^ [15] (approximated to 300 s^−1^) which is associated with an approximate bolus velocity of 0.10 m/s in patients with OD [4,9,17]. 

Another relevant factor to consider when assessing TP viscosity is the effect of salivary amylase during the oral phase of swallowing [18,19,20]. This can have a major impact on viscosity and thus on the therapeutic effect [5]. Salivary amylase is an enzyme produced by three oral glands (parotid, sublingual and submandibular) [19]. The function of this enzyme is to start the oral digestion of carbohydrates and form the alimentary bolus by breaking the chemical structure of the food [19]. An in vitro (artificial saliva) or ex vivo (oral incubation) test reproduces the shear viscosity changes when mixed with saliva in the oral cavity during the oral phase of swallowing. There are also external factors such as fluid temperature or standing time between preparation and ingestion that can also affect the therapeutic effect of TPs [21]. Newtonian fluids do not depend on shear rate (inelastic behavior) but only on temperature [14,22]. They present an indirect effect: Newtonian fluid viscosity decreases with the increment of temperature. In contrast, the multiple dependencies on several variables of non-Newtonian fluids (shear rate, temperature), make them unpredictable and they, therefore, need to be studied individually. Several mathematical approaches have been performed for these fluids, such as the Arrhenius equation [22], which reflects the behavior of non-Newtonian fluids with temperature [23]. Finally, we also need to consider the impact X-ray contrast may have on ShV when performing videofluoroscopy (VFS). VFS is the gold standard diagnostic tool for OD [17], and the procedure consists of taking images while patients swallow boluses of different viscosity in a safety algorithm [17]. VFS is used to assess signs of the impaired safety and efficacy of swallowing, the biomechanics of the swallow response and the therapeutic effect of different strategies. However, the addition of X-ray contrast to the TP solution can modify the ShV and thus the diagnosis of OD [24]. 

The main problem associated with the use of TP is the wide divergence between the viscosity level prescribed, the one tested and the one prepared caused by inaccuracy in measuring shear viscosity and its behavior while being swallowed. A large number of fluid classifications without scientific evidence [2] and based on arbitrary ranges are used around the world such as the viscosity classification proposed by the National Dysphagia Diet (NDD) association [16] or the International Dysphagia Diet Standardisation Initiative (IDDSI) [25]. A study published previously by our group (21) demonstrated that: (a) there is a need to test the viscosity level and to demonstrate the therapeutic effect produced; (b) descriptors used by fluid classification do not correlate with the same viscosity value; (c) composition, preparation method and swallowing factors can alter the shear viscosity of TPs and thus the therapeutic effect. 

The main aim of this research study is to design and validate a rheological protocol to reproduce the oral and pharyngeal factors affecting the therapeutic effect of TP and standardize the viscosity measurements in a scientific and precise manner. Specific aims are: (a) to determine an accurate and homogeneous methodological protocol to prepare TPs; (b) to determine the intra- and inter-laboratory accuracy of the rheological protocol to assess the ShV in SI units in different international laboratories and thus validate it; (c) to characterize the rheological ex vivo and in vitro properties of a xanthan-gum-based TPs (effect of amylase in the oral phase and shear thinning in the pharyngeal phase), and (d) to determine the impact of the X-ray contrast Omnipaque, fluid temperature and lag time from preparation to measurement on the rheological properties of TPs.

## 2. Materials and Methods

### 2.1. Thickening Product (TP)

The TP used for the study is Tsururinko Quickly (TQ; batch 23 March 2021) a xanthan gum-based TP manufactured by Morinaga Milk Industry, Co., ltd, Tokyo, Japan. The nutritional composition is detailed in Table 1. Mineral water was Font D’Or (Vichy Catalan Corporation, Barcelona, Spain), and the X-ray contrast used for VFS was Omnipaque™ (GE Healthcare Bio-Sciences, S.A.U, Madrid, Spain).

### 2.2. Laboratories and Equipment

Validation of the proposed rheological protocol has been performed in 4 laboratories. Laboratory 1: Collaborated company with Morinaga, Japan. MCR 302–Rheometer (Anton Paar, Graz, Austria); Laboratory 2: Health Care and Nutritional Science Institute Morinaga Milk Industry, Co., Ltd., Kanagawa, Japan. MCR 301 Rheometer (Anton Paar, Graz, Austria); Laboratory 3: I+D Laboratory on rheology and alimentary texture of Hospital de Mataró, Mataró, Spain. Haake Viscotester 550 (Thermo Fisher Scientific, Waltham, MA, USA); Laboratory 4: Prefectural University of Hiroshima, Hiroshima, Japan. Haake Rheostress 6000 (Thermo Fisher Scientific, Waltham, MA, USA).

### 2.3. Study Design

**Figure 1:**
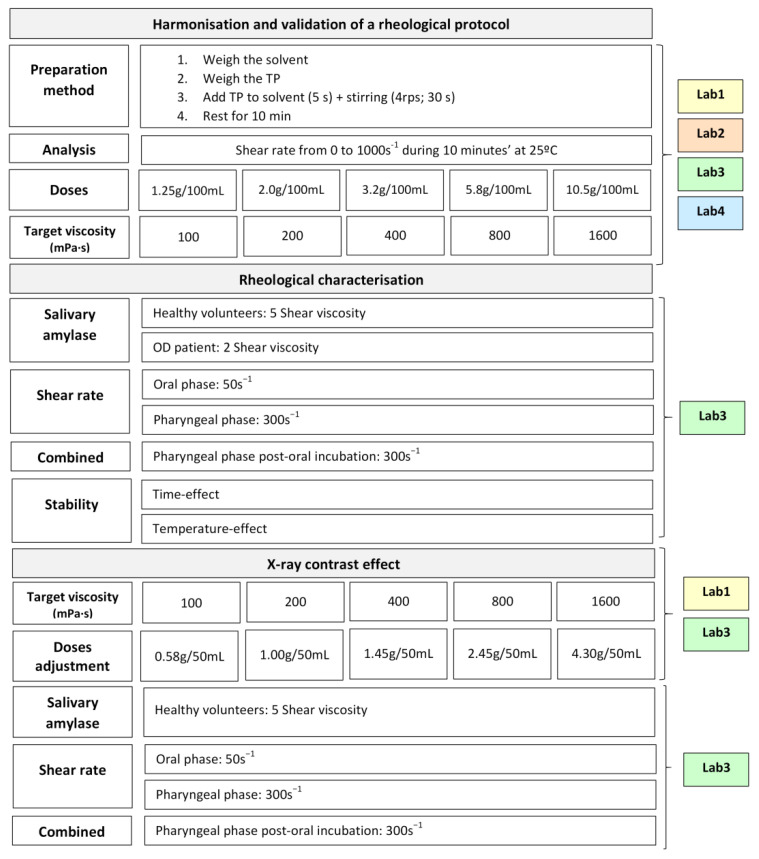
Study design showing the experiments, the measurements and the laboratories involved in the study. Laboratory 1: Collaborated company with Morinaga, Japan; Laboratory 2: Health Care and nutritional science institute, Morinaga Milk Industry, Co., Ltd., Kanagawa, Japan; Laboratory 3: I+D Laboratory on rheology and alimentary texture of Hospital de Mataró, Mataró, Spain; Laboratory 4: Prefectural University of Hiroshima, Hiroshima, Japan. TP: Thickening Product; OD: oropharyngeal dysphagia.

This study includes three different parts: (a) the accuracy, harmonization and validation of a common rheological protocol to assess the shear viscosity of a thickening product in 4 international laboratories; (b) the in vitro rheological characterization (amylase resistance and shear thinning effect) of Tsururinko Quickly (TQ), including the assessment of the effect of fluid temperature and lag time; and (c) the effect of an X-ray contrast on the rheological properties. This study has been approved by the Ethics Committee of the Consorci Sanitari del Maresme under Code 58/19. Figure 1 shows the general design of the study.

### 2.4. Methods

#### 2.4.1. Rheological Protocol

(1) *Harmonization of the preparation protocol*. In order to standardize the preparation method and analysis for the identical rheological protocol to be applied in the four laboratories, the reference laboratory (Lab1) previously assessed the factors that differed, which included: (a) Stirring conditions: rotations per second, stirring speed and time. (a) Stirrer (metallic spatula, 160 mm length plastic spoon and 100 mm length plastic spoon) for all viscosity levels; (b) Container (glass beaker, white plastic cup, clear plastic cup) and; (c) Standing time before measurement (immediately, 10 and 30 min) for 100, 400 and 1600 mPa·s.
(2) *Laboratory Measurements Variability*. Four different facilities: All the laboratories (1, 2, 3 and 4) validated the common rheological protocol to analyze the shear viscosity of the selected TP at different doses (Figure 1). The following harmonized protocol was established: (a) weigh the dissolvent in a clear plastic cup; (b) weigh the TP; (c) add it to the dissolvent over 5 s while stirring at 4 rps with a metallic spatula; (d) continue stirring for 30 s at the same velocity; (e) rest for 10 min; (f) analyze viscosity by increasing the shear rate from 0 to 1000 s^−1^ in a 10 min test at 25 °C. Viscosity measurements were performed in triplicate on three samples for each viscosity level. Daily condition (DC) doses (TP with mineral water) have been used for this test presented in Table 2. Doses have been selected to determine different viscosity levels ranging between 100 and 1600 mPa·s to validate the protocol in a wide range of shear viscosities according to previous studies [5]. These levels allow viscosity behavior to be observed at very low (100–200 mPa·s), medium (400–800 mPa·s) and high (1600 mPa·s) viscosity values. In addition, the therapeutic effect of these viscosity levels for this specific TP is being analyzed in a clinical trial NCT04565587.

#### 2.4.2. Rheological Characterization of TP

The rheological characterization was carried out in Lab3. Briefly, 10 mL of the bolus to be measured was placed in the sensor system gap: MV1 was used for viscosities ranging from 50 to 300 mPa·s and SV1 for higher viscosities. The temperature was set at 25 °C by the ThermoScientific system. Briefly, the bolus exerts resistance to the rotational movement produced by the rotor in an increasing shear rate range from 1 to 1000 s^−1^. The results were analyzed by the software RheoWin (Job Manager^®^ and Data Manager^®^) (Thermo Fisher Scientific, Waltham, MA, USA). 

*Amylase effect.* The oral salivary amylase effect was determined in an ex vivo study [21]. The participants performed an oral incubation, and viscosity was assessed prior and posterior to the incubation in order to reproduce the intra-oral movements and the bolus mixed with saliva when forming or moving the bolus. Briefly, the participant held a bolus of 15 mL in the mouth for 30 s and then spat it out to be analyzed in the viscometer. Amylase testing was always performed in the morning due to the circadian cycle of saliva.

*Shear rate effect.* The shear rate effect was assessed by an in vitro study for the whole swallowing spectrum (1–1000 s^−1^) [11,21]. To simulate the effect of the shear rate affecting the bolus when swallowing, the viscosity was determined at the shear rate during the oral phase (50 s^−1^) and the pharyngeal phase (300 s^−1^) [5].

*Combined effect of swallowing factors.* ShV behavior was also measured when submitted to two swallowing factors: salivary amylase in the oral phase and shear rate in the pharyngeal phase. Viscosity at 300 s^−1^ was determined after oral incubation. 

*Time effect.* The effect of the standing time between the preparation and measurement of the TP viscosity is an important factor to be determined. To assess the effect of time for this specific TP, two DC solutions (200 and 800 mPa·s) were analyzed at several timings: at the moment when the solution is prepared and 30, 60, 90 and 120 min later. 

*Temperature effect.* The temperature effect on ShV was analyzed in individual experiments by increasing the temperature range of the TP solution in steps of 5 °C from 25 to 40 °C.

#### 2.4.3. Effect of X-ray Contrast on the ShV of TP

Solutions were adapted for the addition of X-ray contrast (Omnipaque^TM^; GE Healthcare Bio-Sciences, S.A.U, Chicago, IL, USA.) to perform VFS for the diagnosis of OD (VFS doses). The final volume was assessed at 50 mL with a 1:1 proportion of water and X-ray contrast. Doses of TQ (g/100 mL water) were adjusted to provide the expected viscosity by the reference Laboratory (Lab1) and the Health Care Centre (Lab3) where OD examinations are performed. The amylase effect during oral incubation in healthy volunteers and shear thinning were assessed as well as the combined effects of both.

### 2.5. Participants

*Healthy volunteers.* Eight healthy volunteers were recruited at the Hospital de Mataró, Catalonia, Spain to participate in the study to assess the effect of α-salivary amylase during the oral incubation of each viscosity level assessed. The participants had to incubate two viscosity boluses per day (1. DC doses; 2. VFS doses). This part lasted 5 days for each participant. The main inclusion criteria were to be older than 18 years and able to sign the informed consent. The main exclusion criteria were not accomplishing the inclusion criteria, suffering from Sjögren syndrome, taking drugs that affect salivation and irritation or inflammation of the oral cavity. 

The number of participants was calculated to detect a difference equal to or higher than 160 mPa·s, accepting an alpha risk of 0.05 and a beta risk of 0.1 in bilateral contrast, so 8 participants were needed. A standard deviation of 100 is assumed with a loss of 0.2. The study design and patient recruitment were approved by the Ethics Committee of the Consorci Sanitari del Maresme with code 58/19.

### 2.6. Measurements and Data Analysis

To assess the effect of the shear rate, viscosities at 50 s^−1^ and 300 s^−1^ at 25 °C were interpolated from the regression line obtained from the shear rate range from 1 to 1000 s^−1^. Viscosity flow curves were fitted to the Ostwald-de Waele model or Power Law (Equation (1)) and the index flow (*n*) and consistency (*K*) were obtained for each viscosity level to assess the viscosity (*η*) behavior of the TP at the shear rate studied (*γ*). The index flow describes the relationship between viscosity and shear rate and divides the fluids’ behavior into Pseudoplastic (shear thinning) or Dilatant (shear thickening). The consistency factor indicates the fluid viscosity at the specific shear rate of 1.

Log *η* = (*n* − 1) log (*γ*) + *K* (1)

**Figure 2:**
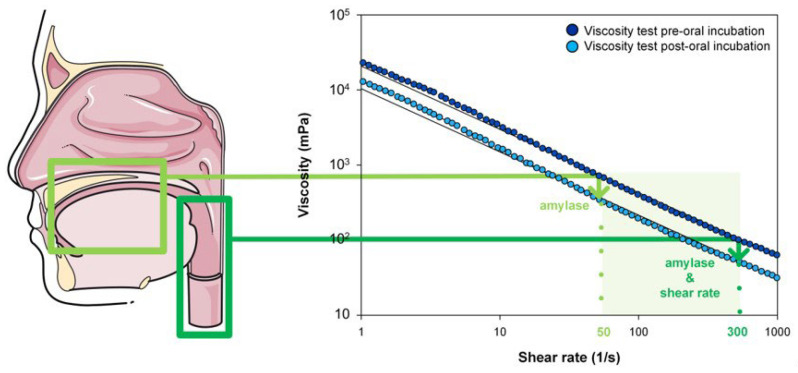
Salivary amylase and shear thinning effect analysis performed in a viscosity curve. Light green corresponds to the oral phase (salivary amylase effect at a shear rate of 50 s^−1^). Dark green corresponds to the increment of shear rate from the oral to the pharyngeal phase (from 50 s^−1^ to 300 s^−1^), including the salivary amylase effect.

Viscosity flow curves are represented by a Cartesian coordinate system where the dependent variable is the shear rate range represented in s^−1^ and the independent variable shows the relative viscosity in mPa. The shear rate effect was calculated by the variations in the apparent viscosity between the value at 50 s^−1^ and at 300 s^−1^. The amylase effect on TQ was determined by an analysis of the shear viscosity after an oral incubation in HV and patients with OD by calculating the differences in apparent viscosity value between the sample at 50 s^−1^ (reference sample) and the incubated sample for each level of viscosity. The viscosity affection by both swallowing factors (amylase and shear rate) was also calculated by the difference between the reference sample and the apparent viscosity at 300 s^−1^ after oral incubation (Figure 2).

The variability between the laboratory values has been presented in mean ± CV. Quantitative data have been used to describe the decrease in viscosity through salivary amylase or shear rate and are presented as a percentage of absolute frequencies. The significance of the viscosity decrease by those factors has been analyzed by a paired *t*-test. Continuous data are presented as mean ± standard deviation (SD). The statistical tests applied to assess the temperature and time effect were the non-parametric ANOVA test (Kruskal–Wallis) to compare all the groups and the U-Mann–Whitney test to assess the 1:1 difference. Significance was considered at *p* < 0.05.

## 3. Results

### 3.1. Design and Validation of a Common Protocol to Standardize Rheological Measurements

*Harmonization of the preparation protocol* (Table 3). (a) Stirring conditions: a homogeneous protocol was selected to be performed in the four different labs; TP was added to the solvent in 5 s, the speed of stirring was determined at 4 rps and the stirring time was assessed at 30 s; (b) Stirrer: a metallic spatula was selected for presenting the lowest viscosity variability ranging between 1.2 and 5.8% for all viscosity levels; in contrast, both plastic spoons obtained a higher variability, ranging from 2.4 to 18.3% and 3.9 to 14.6% for the 160 mm and 100 mm length spoons, respectively; (c) Container: the variability for each viscosity level was similar for the glass beaker (1.8–6.3%) and the clear plastic cup (1.9–5.7%). The white plastic cup presented the highest viscosity variation (5.2–12.0%). A clear plastic cup was selected for this study; (d) Standing time before measurement: the thickened viscosities varied widely when assessed immediately after preparation when prepared in a glass beaker (2.9–12.8%) and in a clear plastic cup (3.1–12.9%), and the variability was reduced after 10 min. Leaving the preparations standing for 10 and 30 min after mixing reduced the variation to a maximum of 6.6%. All the data are presented in Table 3.

*Laboratory variability* (Table 4). The mean intralaboratory coefficient of variation on the measurements at all target ShV levels (100–1600 mPa·s) was very low: 0.9% (Lab1); 3.9% (Lab2); 2.7% (Lab3); 2.9% (Lab4), and similar for all viscosity levels. The global mean interlaboratory variability was higher: 9.34%, it but did not exceed 10% differences for the viscosity levels tested except for the lowest, and this was caused by measurements only in Lab3 at 100 mPa·s. No significant differences in viscosity values were obtained for the ShV levels assessed except for 100 mPa·s (*p* = 0.038) caused by the measurements in Lab3. Table 4 presents the mean viscosity value for each level determined for the various facilities. Finally, for the rheological characterization, (Lab3) the dose to achieve the 100 mPa·s level was increased to 1.45 g in order to reduce the variation between the analyzed viscosity values and the target viscosity which was 19% for 1.25 g/100 mL and reduced to 7.70% when using 1.45 g.

### 3.2. Rheological Characterization

*Amylase effect.* The mean age of the volunteers was 32 ± 3.76 years, and 25% were females. In healthy volunteers, the amylase effect produced a viscosity decrease ranging from −0.4 (increase) to 16%. The mean ShV values are presented in Table 5 for each dose of the TP assessed. The mean results of HV are presented in Table 5 and Figure 2. 

*Shear rate effect.* This specific TP, TQ, presents an index flow ranging from 0.16–0.29 which confirms the pseudoplastic behavior of the fluid (*n* < 1) (Table S1). Shear thinning at 300 s^−1^ caused a significant reduction in the apparent viscosity ranging between 77 and 78% (*p* < 0.05 vs. 50 s^−1^), which is in line with xanthan gum-based TP previously determined [21].

**Figure 3:**
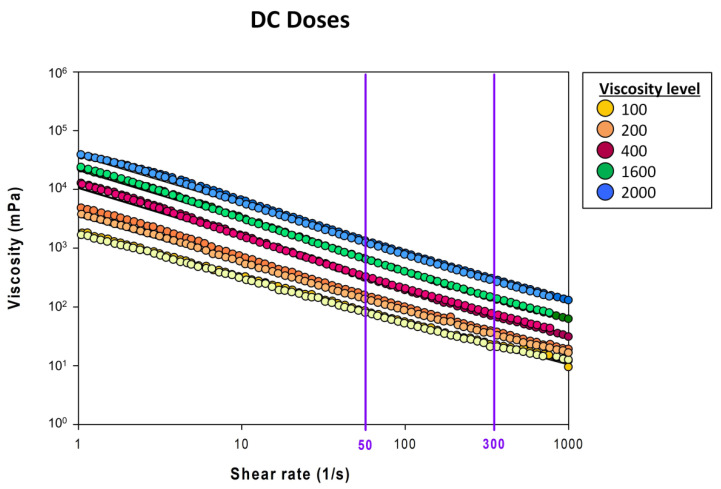
Viscosity curves from a shear rate range of 1–1000 s^−1^ for daily condition doses before and after oral incubation in healthy volunteers. Dark colors correspond to viscosity levels pre-oral incubation. Soft colors correspond to viscosity post-oral incubation. Purple lines mark the two main shear rate landmarks (50 and 300 s^−1^) during deglutition in patients with oropharyngeal dysphagia.

*Combined effect of oral and pharyngeal swallowing factors.* The combined effect of salivary amylase (oral phase) and shear rate (pharyngeal flow) produced a decrease in viscosity ranging from 75–79% in HV (Table 5, Figure 3). The combination of both parameters did not present a summatory effect but a slight increase in the impact on ShV values in a significant manner for all viscosity levels assessed.

**Figure 4:**
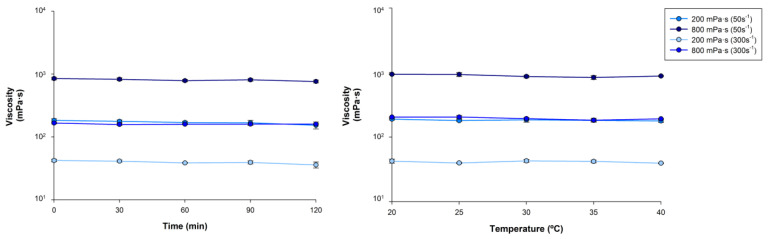
Viscosity values (mean ± SD at 50 and 300 s^−1^) by increasing time from 0 to 120 min (**right**) and temperature from 20 to 40 °C (**left**) for 200 and 800 mPa·s levels for daily condition doses.

*Time effect.* A variability of 16% was observed at the 200 mPa·s level for the ShV values assessed at the five different times (Table S2). At 800 mPa·s, the variability in the ShV values was reduced to a maximum of 10%. No significant differences appeared between the ShV values assessed for multiple comparisons, nor between all values. After 120 min, ShV experienced a decrease ranging from 9 to 17% at 200 mPa.s and from 3 to 10% at 800 mPa.s (Figure 4).

*Temperature-effect.* Increasing the temperature by 5 °C caused a viscosity variation ranging from 1.9 to 5.1% at 200 mPa·s at 50 s^−1^ (*p* > 0.05) and between 0.74 and 6.9% (*p* > 0.05) at 800 mPa·s at 50 s^−1^. The maximal temperature assessed (40 °C) caused a decrease in viscosity between 6–7% for both ShV levels assessed. The viscosity values assessed for each temperature level and for both viscosity levels are presented in Table S3. The ShV values presented no significant differences by increasing the temperature for the whole temperature frame nor by multiple comparisons (Figure 4).

### 3.3. Effect of the X-ray Contrast Omnipaque on the Rheological Properties 

*Dose adaptation.* Solutions with X-ray contrast in water (1:1 *vol*/*vol*) were adapted to the volume normally used for VFS in Lab3 (50 mL) to achieve the target ShV. The doses were calculated to achieve the same viscosity levels obtained by the DC doses previously described. Table 6 shows the dose of TQ necessary to obtain each target viscosity level when using the X-ray contrast as a solvent. The doses needed to achieve each ShV level were slightly lower than for the DC doses (Table 7) varying between: 20.0, 0, 9.4, 15.5 and 18.1% for 100, 200, 400, 800 and 1600 mPa·s, respectively.

The VFS doses presented similar results at the shear rate of 50 s^−1^ between Lab1 and Lab3. The variability was calculated for each viscosity: 17.5%, 2.5%, 0.7%, 2.3% and 4.6% for 100, 200, 400, 800 and 1600 mPa·s, respectively. The maximal difference appeared for the lowest viscosity level (100 mPa·s) according to the previous results presented above. Similar variations appeared for viscosities at 300 s^−1^ (Table 7). The VFS doses were calculated in order to obtain no significant differences in the ShV according to the DC viscosity doses. The viscosity curve is presented in Figure S2.

*Amylase effect.* Oral incubation caused a decrease in viscosity in the healthy volunteers ranging from 11 to 20% for VFS doses. A slight increase was seen after oral incubation for the lowest viscosity level (100 mPa·s). Table 8 presents the results for the VFS doses after oral incubation. Significant differences only appeared for the highest levels assessed (800 and 1600 mPa·s). No significant differences appeared when comparing the ShV values post-oral incubation between the DC doses and the VFS doses.

*Shear rate effect.* The index flow ranged between 0.16 and 0.32 (Table S4). The shear thinning effect caused a viscosity decrease from 50 s^−1^ to 300 s^−1^ of 70–78% for VFS. Significant differences appeared between the ShV at 50 s^−1^ and 300 s^−1^ for all the levels tested (*p* < 0.0001). Similar results have been obtained for the VFS and DC doses. 

*Combined effect.* The combined effect of salivary amylase (oral phase) and shear rate (pharyngeal flow) produced a viscosity decrease ranging from 69 to 83% with significant differences for all viscosity levels. No significant differences appeared when comparing the ShV values post-oral incubation for the DC doses and the VFS doses. 

## 4. Discussion

The main conclusions of this study arise from two separate groups of results: the first demonstrated that, under the same conditions, different international laboratories with different equipment can obtain very similar shear viscosity results (actual measured viscosity), supporting the implementation of a common protocol and the use of SI units (mPa·s) to accurately report the viscosity of TPs for OD patients. We developed an in vitro/ex vivo rheological protocol that reproduces the oral (amylase effect) and pharyngeal conditions (shear thinning associated with pharyngeal bolus flow) that can affect the therapeutic effect of TP. The second group of results came from applying this new rheological protocol to a particular TP, in this case, TQ, to fully characterize its rheological properties. This is because we are currently assessing the therapeutic properties of TQ in a clinical trial with OD patients registered on the Clinical Trials Gov website with code: NCT04565587. The four laboratories accurately measured the rheological properties with very little difference between them and found that TQ was highly resistant to salivary amylase in healthy people and OD patients during the ex vivo assessment of the oral phase and behaved as a non-Newtonian fluid with a moderate shear thinning effect during the in vitro conditions for the pharyngeal phase. The viscosity values presented in this study correspond to the actual measured viscosity in SI units. 

The known therapeutic effect of TPs is linked to the shear viscosity obtained [5] and has been extensively studied in several phenotypes of patients with OD [3,4,6,9] by our research group. By performing shear viscosity dose–response curves of TPs [5,6], we obtained two main results: the therapeutic range and the optimal shear viscosity levels. Those results showed the maximal viscosity to be below 1000 mPa·s at 50 s^−1^ and that by using only two shear viscosity levels, we can manage more than 80% of the OD population. However, commercially available TPs still recommend three thickness levels (randomly selected) and describe them with qualitative descriptors that also differ [21,26]. In addition, essential information affecting the therapeutic effect of TPs, such as the quantitative composition, amylase effect or shear thinning behavior, is not reported on the label or prospectus although they can greatly alter shear viscosity, especially in MS TPs, and this represents a risk to patients [21]. This study shows the path to move forward from qualitative to quantitative measurements in the use of TPs and their rheological characterization. As we have observed in the results of this study, the preparation method (container, stirrer, stirring method and standing time) can alter the shear viscosity of TPs. However, by applying this standardized protocol we have proved that we can control the potential variability of these factors. In addition, differences due to intra and inter-laboratory variability (using different measurement equipment) were maintained below 5% and 10%, respectively, which means that this protocol is optimal to be applied worldwide. This protocol has been developed to be applied by specialized nutritional companies manufacturing TPs (mainly considered under the regulations of food for medical purposes) and also in rheology labs to identify the real viscosity levels prescribed to patients with OD and the therapeutic profile of each TP during the swallowing process. This allows clinical experts to explore, in an accurate and reproducible manner, the therapeutic effect of TPs by observing whether the rheologic properties of thickening products are maintained during swallowing. There is a need to standardize not only the viscosity levels selected to treat patients but also the methodology to prepare them. A normalized procedure to measure viscosity has been added as Supplementary Material (Supplementary Information S1).

With this study, we propose a rheological protocol to accurately determine the behavior of TPs by simulating oral and pharyngeal forces and other external factors such as the preparation method, temperature and timing, all of which can impact shear viscosity and, therefore, the therapeutic effect of a TP. This study can also be applied to assess the viscosity behavior in different alimentary fluids to determine whether the viscosity of the products is maintained or, in contrast, need to be adjusted depending on the solvent. First, the shear viscosity is assessed in the whole shear rate frame of an oral and pharyngeal swallow (0–1000 s^−1^; [15]) and the viscosity interpolated at the two main shear rate landmarks: 50 s^−1^ [16,27] and 300 s^−1^ [15], simulating the shear forces at the oral and pharyngeal phase, respectively in laboratory conditions. Pharyngeal shear rate approximation has been explained elsewhere [21]. Other methods such as the Gothenburg throat [28] can also be used to predict and simulate the behavior of TPs during swallowing. However, the ex vivo analysis reflects, in a very accurate and specific manner, exactly what occurs during the bolus preparation according to the swallowing function of different phenotypes. This is the reason why we propose oral incubation rather than artificial saliva, as it allows us to assess and compare the differences between population groups (young, older and patients with OD and other conditions). 

A previous study published by our group demonstrated that TPs can be divided into three groups according to their rheological patterns [21]: the first group included TPs composed of modified starch and highly affected by salivary amylase (80–100%) and shear thinning ranging from 50–70%; the second group included TPs with a mixture of MS and gums that was less affected by salivary amylase (30–40%) and the third group composed by XG included amylase-resistant TPs with a maximal decrease of 20%. The latter two groups presented shear thinning behavior ranging from 60 to 77%. These differences between the TPs show the need to analyze how each TP behaves in order to prescribe the one most suited to each patient. As we have demonstrated, a full rheological characterization is possible and demonstrates fluid behavior when being swallowed in a scientific, reproducible and accurate manner. The studied TP, TQ, presented optimal characteristics due to its resistance to salivary amylase [21] but also due to its high stability to the increment of temperature and standing time, factors which are critical in the daily use of TPs. Another aspect worthy of comment is the quantity of TP needed to obtain the various shear viscosity levels. Compared to other TPs [21], the grams of TQ needed to obtain the shear viscosity levels are similar to other XG-based TPs and far fewer than MS-based TPs. Another point to take into account with TPs is the viscosity obtained after the addition of X-ray contrast to perform a VFS. VFS is the gold standard diagnostic technique for OD, and in order to perform a VFS, X-ray contrast is used to visualize the bolus transit. An accurate characterization of the impact of the X-ray contrast on the shear viscosity is important. This study has shown that the addition of X-ray contrast to this specific TP caused no significant impact on the shear viscosity, which makes the product also optimal to study swallowing impairments with VFS. 

TPs are included in the group of Food for Special Medical Purposes (FSMPs), and they are intended to manage OD [7]. For this reason and all of the factors explained above, an international and scientific manner of describing TP rheological properties is of great importance and urgency. As presented in this study, current technology enables the rheological characteristics of TP to be determined and objectively measured and, if described with the International System of Units (SI) [12], they are comparable and reproducible worldwide. The appropriate labeling of products according to the properties presented in this study in SI units would help reduce the risks patients are exposed to and would also help healthcare professionals in managing individual patients and providing standardized care. 

This study also has some limitations including: (1) in this study, only one TP was assessed. However, the rheological protocol can be applied to any and all TPs; (2) other rheological factors such as extensional properties or tribology could affect the therapeutic effect and were not assessed in this study; (3) low specificity to measure low viscosities (100 mPa·s) with the rotors used in Lab3. 

In summary, we have developed a rheological protocol that can be applied around the world and simulates TP behavior during swallowing by patients with OD in an accurate and reproducible manner. We believe that, step by step, we can move from the qualitative approach to the quantitative and SI system which will improve not only the safety of our patients but also the quality of the care provided by healthcare professionals.

## 5. Conclusions

This study shows that different laboratories worldwide can obtain very accurate and similar shear viscosity measurements of thickening products using this protocol which uses scientific, universal SI units (mPa·s). In addition, our protocol accurately reproduces the oral and pharyngeal factors affecting the therapeutic effect of thickening products. We claim labels of thickening products should include viscosity in mPa·s at 50 s^−1^ and the effect of salivary alpha-amylase and pharyngeal shear thinning as main factors affecting the therapeutic effect and mode of action of thickening agents to improve the clinical management of patients with oropharyngeal dysphagia.

## Figures and Tables

**Table 1:** Batch used for the study, nutritional composition and ingredients of Tsururinko Quickly.

**Ingredients**	Dextrin, xanthan gum, calcium lactate, trisodium citrate	
**Nutritional Composition**	Energy	270 kcal
	Protein	0.5 g
	Lipid	0 g
	Carbohydrates	88.9 g
	Fiber	21.9 g
	Sodium	960 mg
	Potassium	980 mg
	Phosphorus	30 mg
	Ash	4.5 g
	Water	6.1 g

**Table 2:** Doses used to validate the common rheological protocol.

DC Doses (g/mL)		
Tsururinko Quickly (g)	Final Volume (mL)	Solvent
1.25	100	Mineral water
2	100	Mineral water
3.2	100	Mineral water
5.8	100	Mineral water
10.5	100	Mineral water

DC: Daily condition.

**Table 3:** Stirring conditions tested by the reference lab to harmonize the rheological protocol between laboratories: stirrer, container and standing time.

Stirrer						
Targeted Viscosity at 50 s^−1^ (mPa·s)	Metallic Spatula 180 mm		Plastic Spoon 160 mm		Plastic Spoon 100 mm	
	Viscosity at 50 s^−1^ (mPa·s)	Variability (%)	Viscosity at 50 s^−1^ (mPa·s)	Variability (%)	Viscosity at 50 s^−1^ (mPa·s)	Variability (%)
100	97.6 ± 3.3	1.2–5.8	107.0 ± 10.8	2.4–18.3	109.1 ± 9.0	3.9–14.6
200	203.8 ± 6.0		212.0 ± 5.4		202.3 ± 9.1	
400	400.4 ± 4.2		402.2 ± 8.6		388.2 ± 8.0	
800	802.8 ± 7.1		767.8 ± 31.1		811.6 ± 21.6	
1600	1602.4 ± 10.1		1572.4 ± 19.6		1595.5 ± 31.7	
**Container**						
**Targeted Viscosity** **at 50 s^−1^ (mPa·s)**	**Glass Beaker**		**White Plastic Cup**		**Clear Plastic Cup**	
	**Viscosity at 50 s^−1^ (mPa·s)**	**Variability (%)**	**Viscosity at 50 s^−1^ (mPa·s)**	**Variability (%)**	**Viscosity at 50 s^−1^ (mPa·s)**	**Variability (%)**
100	98.1 ± 3.5	1.8–6.3	107.6 ± 4.7	5.2–12.0	101.9 ± 3.3	1.9–5.7
400	398.4 ± 3.90		401.0 ± 25.6		407.4 ± 5.0	
1600	1597.0 ± 18.1		1555.3 ± 42.1		1573.1 ± 16.0	
**Standing Time–Glass Beaker**						
**Targeted Viscosity** **at 50 s^−1^ (mPa·s)**	**0 min**		**10 min**		**30 min**	
	**Viscosity at 50 s^−1^ (mPa·s)**	**Variability (%)**	**Viscosity at 50 s^−1^ (mPa·s)**	**Variability (%)**	**Viscosity at 50 s^−1^ (mPa·s)**	**Variability (%)**
100	100.9 ± 6.2	2.9–12.8	100.0 ± 3.0	1.4–5.6	98.4 ± 3.0	1.3–5.4
400	394.3 ± 29.6		403.7 ± 6.8		398.5 ± 4.1	
1600	1620.3 ± 24.7		1588.3 ± 13.0		1595.1 ± 12.0	
**Standing Time–Clear Plastic Cup**						
**Targeted Viscosity** **at 50 s^−1^ (mPa·s)**	**0 min**		**10 min**		**30 min**	
	**Viscosity at 50 s^−1^ (mPa·s)**	**Variability (%)**	**Viscosity at 50 s^−1^ (mPa·s)**	**Variability (%)**	**Viscosity at 50 s^−1^ (mPa·s)**	**Variability (%)**
100	102.2 ± 6.4	3.1–12.9	100.8 ± 3.2	1.3–6.0	99.4 ± 3.4	1.4–6.6
400	415.1 ± 29.6		401.4 ± 7.8		407.4 ± 5.0	
1600	1670.1 ± 27.4		1644.3 ± 11.1		1605.4 ± 12.5	

**Table 4:** Mean values and the coefficient of variation from the triplicates of each viscosity level performed by the four labs. Variations within measurements in the same facility are shown as well as variations between facilities for each viscosity level; * *p* < 0.05.

Dosage TP (g/100 mL)	Viscosity (mPa·s) at 50 s^−1^								Mean Interlaboratory Variability (%)	*p*-Value
	Lab1		Lab2		Lab3		Lab4			
	Mean (mPa·s)	±CV (%)	Mean (mPa·s)	±CV (%)	Mean (mPa·s)	±CV (%)	Mean (mPa·s)	±CV (%)		
1.25	101	±2.6	105	±4.9	81	±5.2	101	±4.3	22.9	*
2	205	±0.09	199	±4.5	190	±1.3	197	±2.3	7.3	0.08
3.2	403	±0.85	396	±4.1	386	±0.97	381	±3.2	5.5	0.13
5.8	805	±0.38	818	±3.0	768	±2.6	797	±3.4	6.1	0.11
10.5	1602	±0.35	1601	±2.8	1552	±3.5	1632	±1.3	4.9	0.16

*: *p* < 0.05.

**Table 5:** Shear viscosity decrease caused by both swallowing factors (salivary amylase in the oral phase and shear thinning in the pharyngeal phase) in healthy subjects.

Healthy Volunteers (*n* = 8)			
Targeted Viscosity (mPa·s) at 50 s^−1^	Viscosity (mPa·s) at 50 s^−1^ Mean ± SD	Amylase Effect (%)	*p*-Value
100	93.1 ± 6.5	5.9	0.99
200	160.0 ± 7.3	15.6	0.75
400	355.5 ± 23.7	7.1	0.81
800	771.8 ± 42.0	−0.37	>0.99
1600	1449.0 ± 72.8	6.7	***
**Targeted Viscosity (mPa·s) at 50 s^−1^**	**Viscosity at 300 s^−1^** **Post-Oral Incubation** **(mean ± SD)**	**Shear Rate** **+ Amylase Effect (%)**	***p*-Value**
100	25.2 ± 1.7	74.5	****
200	39.1 ± 1.8	79.4	****
400	78.8 ± 4.1	79.4	****
800	168.1 ± 9.0	78.1	****
1600	329.3 ± 20.0	78.8	****

*** *p* < 0.001 and **** *p* < 0.0001 vs. pre-oral incubation shear viscosity values.

**Table 6:** Doses used to mix the videofluoroscopy doses.

VFS Doses (g/mL)			
Targeted Viscosity (mPa·s) at 50 s^−1^	Tsururinko Quickly (g)	Final Volume (mL)	Dissolvent (mL)
100	0.58	50	1:1 (water:Omnipaque)
200	1	50	1:1 (water:Omnipaque)
400	1.45	50	1:1 (water:Omnipaque)
800	2.45	50	1:1 (water:Omnipaque)
1600	4.3	50	1:1 (water:Omnipaque)

VFS: videofluoroscopy.

**Table 7:** Viscosity assessment between Lab1 (reference lab) and Lab3 (Healthcare center for the CT) for the VFS doses.

Targeted Viscosity at 50 s^−1^ (mPa·s)	Dosage (g/50 mL)	Average Viscosity at 50 s^−1^ (mPa·s), *n* = 3		Variations Within Facilities (%, *n* = 3)		Variations between Facilities (%)	Lab1-Targeted Viscosity (%)	Lab3-Targeted Viscosity (%)
		Lab1	Lab3	Lab1	Lab3			
100	0.58	114	94	1.0	7.2	17.5	14	6
200	1	239	233	0.54	6.7	2.5	19.5	16.5
400	1.45	446	443	0.68	12.0	0.7	11.5	10.8
800	2.45	833	852	1.0	8.4	2.3	4.1	6.5
1600	4.3	1598	1672	0.6	3.7	4.6	0.13	4.5
**Targeted Viscosity** **at 50 s^−1^** **(mPa·s)**	**Dosage (g/50 mL)**	**Average Viscosity at 300 s^−1^ (mPa·s, *n* = 3)**		**Variations Within Facilities (%, *n* = 3)**		**Variations Between Facilities (%)**		
		**Lab1**	**Lab3**	**Lab1**	**Lab3**			
100	0.58	32	28	0.77	1.9	12.5		
200	1	58	57	0.52	4.23	1.7		
400	1.45	99	100	0.5	13.4	1.0		
800	2.45	178	187	0.82	9.2	5.1		
1600	4.3	349	384	0.63	5.6	10.0		

**Table 8:** Shear viscosity decrease caused by both swallowing factors (salivary amylase in the oral phase and shear thinning in the pharyngeal phase) in healthy subjects.

**Healthy Volunteers (*n* = 8)**			
**Targeted Viscosity (mPa·s) at 50 s^−1^**	**Viscosity (mPa·s) at 50 s^−1^** **Mean ± SD**	**Amylase Effect** **(%)**	***p*-Value**
**100**	99.0 ± 11.6	−4.47 (increment)	>0.99
**200**	187.0 ± 21.4	19.7	0.98
**400**	355.5 ± 23.7	19.8	0.75
**800**	731.5 ± 33.1	14.1	**
**1600**	1476.3 ± 115.9	11.7	****
**Targeted Viscosity (mPa·s) at 50 s^−1^**	**Viscosity at 300 s^−1^** **Post-Oral Incubation** **(Mean ± SD)**	**Shear Rate** **+ Amylase Effect (%)**	***p*** **-Value**
**100**	28.9 ± 3.1	69.5	****
**200**	47.5 ± 5.0	79.6	****
**400**	78.8 ± 4.1	83.1	****
**800**	161.9 ± 6.3	82.2	****
**1600**	331.6 ± 29.0	80.5	****

** *p* < 0.01 and **** *p* < 0.0001 vs. pre-oral incubation shear viscosity values.

## Data Availability

Not applicable.

## Supplementary Materials

The following supporting information can be downloaded at: https://www.mdpi.com/article/10.3390/nu14235028/s1, Table S1: Index flow, consistency and shear thinning effect from 50 to 300 s^−1^ for daily condition (DC) doses; Table S2: Viscosity values (mean ± SD) by standing time for 200 and 800 mPa·s; *p* > 0.05 (ns); Table S3: Viscosity values (mean ± SD) by increasing temperature from 20 °C to 40 °C for 200 and 800 mPa·s levels for daily conditions doses; *p* > 0.05 (ns); Table S4. Index flow, consistency and shear thinning effect from 50 to 300 s^−1^ for VFS doses; Figure S1: Inter-laboratory variability for each shear viscosity level tested (100, 200, 400, 800 and 1600 mPa·s); Figure S2: Viscosity curves from a shear rate range of 1–1000 s^−1^ for videofluoroscopy doses before and after oral incubation in healthy volunteers. Dark colors correspond to viscosity levels pre-oral incubation. Soft colors correspond to viscosity post-oral incubation. Purple lines mark the two main shear rate landmarks (50 and 300 s^−1^) during deglutition in patients with oropharyngeal dysphagia; Supplementary Information S1: Normalized protocol to determine shear viscosity for thickening products.
