# Supplementary material for: Proposal for a Standard Protocol to Assess the Rheological Behavior of Thickening Products for Oropharyngeal Dysphagia
Source: Nutrients. 2022 Nov 25;14(23):5028. doi: 10.3390/nu14235028 (PMC9738203; doi:10.3390/nu14235028)
Supplement: Supplementary file 1 [file nutrients-14-05028-s001.zip › nutrients-2025852 - supplementary.pdf]

nutrients MDPI

Supplementary Materials

Table S1. Index flow, consistency and shear thinning effect from 50 to 300 s^{-1} for daily condition (DC) doses.

DC doses			
**Target viscosity (mPa·s) at 50 s^{-1}**	**Index flow (*n*)**	**Consistency (K)**	**Shear thinning (%)**
**100**	0.29	3.1	77.1
**200**	0.19	3.7	76.5
**400**	0.16	4.0	78.0
**800**	0.16	4.3	77.8
**1600**	0.17	4.6	77.2

DC: Daily condition.

Table S2. Viscosity values (mean±SD) by standing time for 200 and 800 mPa·s; $p > 0.05$ (ns).

	200 mPa·s (Target viscosity at 50 s^{-1})			800 mPa·s (Target viscosity at 50 s^{-1})		
**Standing time (min)**	**Viscosity (mPa·s) at 50 s^{-1} (mean±SD)**	**Differences 0-120 min (%)**	***p*-value**	**Viscosity (mPa·s) at 50 s^{-1} (mean±SD)**	**Differences 0-120 min (%)**	***p*-value**
**0**	183.3±11.0	16.6	0.46	848.2±38.4	10.2	0.054
**30**	177.0±9.0			827.1±42.7		
**60**	169.5±8.3			784.9±32.7		
**90**	167.5±15.3			809.4±38.4		
**120**	152.9±19.1			762.1±39.4		
	**200 mPa·s (Target viscosity at 50 s^{-1})**			**800 mPa·s (Target viscosity at 50 s^{-1})**		
**Standing time (min)**	**Viscosity (mPa·s) at 300 s^{-1} (mean±SD)**	**Differences 0-120 min (%)**	***p*-value**	**Viscosity (mPa·s) at 300 s^{-1} (mean±SD)**	**Differences 0-120 min (%)**	***p*-value**
**0**	42.1±2.0	14.9	0.051	166.0±35.3	3.9	0.40
**30**	41.1±1.5			157.7±5.9		
**60**	38.5±0.3			159.2±3.1		
**90**	39.2±2.2			159.4±2.3		
**120**	35.8±3.9			159.6±2.9		

Table S3. Viscosity values (mean±SD) by increasing temperature from 20ºC to 40ºC for 200 and 800 mPa·s levels for daily conditions doses; $p>0.05$ (ns).

	200 mPa·s (Target viscosity at 50 s^{-1})			800 mPa·s (Target viscosity at 50 s^{-1})		
**Temperature (ºC)**	**Viscosity (mPa·s) at 50 s^{-1} (mean±SD)**	**Differences 0-40ºC (%)**	***p*-value**	**Viscosity (mPa·s) at 50 s^{-1} (mean±SD)**	**Differences 0-40ºC (%)**	***p*-value**
**20**	190.2±6.5	6.3	0.17	848.2±38.4	5.9	0.09
**25**	180.5±5.8			827.1±42.7		
**30**	185.3±15.3			784.9±32.7		
**35**	181.6±5.6			809.4±38.4		
**40**	178.1±8.9			762.1±39.4		
	**200 mPa·s (Target viscosity at 50 s^{-1})**			**800 mPa·s (Target viscosity at 50 s^{-1})**		
**Temperature (ºC)**	**Viscosity (mPa·s) at 300 s^{-1}**	**Differences 0-40ºC (%)**	***p*-value**	**Viscosity (mPa·s) at 300 s^{-1}**	**Differences 0-40ºC (%)**	***p*-value**

	(mean±SD)			(mean±SD)		
**20**	42.4±3.1			166.03±5.34		
**25**	39.7±0.9			157.70±5.90		
**30**	42.8±2.2	7.0	0.20	159.23±3.10	5.8	0.11
**35**	42.0±1.9			159.43±2.27		
**40**	39.4±0.3			159.57±2.87		

Table S4. Index flow, consistency and shear thinning effect from 50 to 300 s^{-1} for VFS doses.

VFS doses			
**Target viscosity at 50 s^{-1} (mPa·s)**	**Index flow (*n*)**	**Consistency (K)**	**Shear thinning (%)**
**100**	0.32	3.1	70.2
**200**	0.23	3.7	75.5
**400**	0.16	4.1	77.4
**800**	0.16	4.4	78.1
**1600**	0.18	4.6	77.0

VFS: videofluoroscopy.

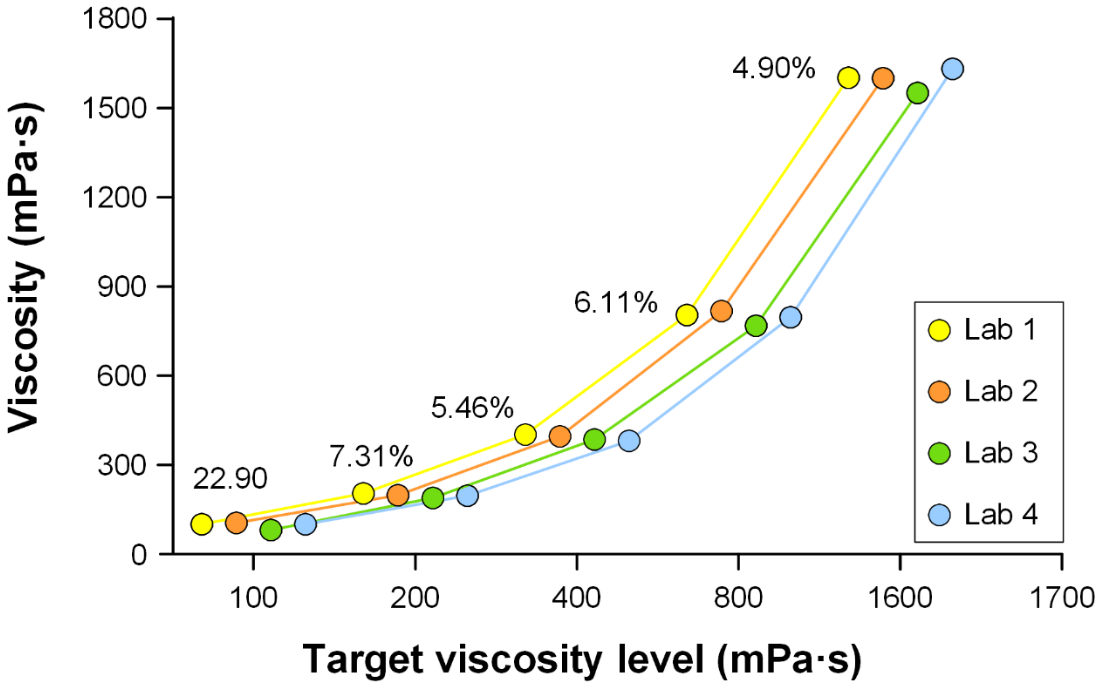

Figure S1. Inter-laboratory variability for each shear viscosity level tested (100, 200, 400, 800 and 1600 mPa·s).

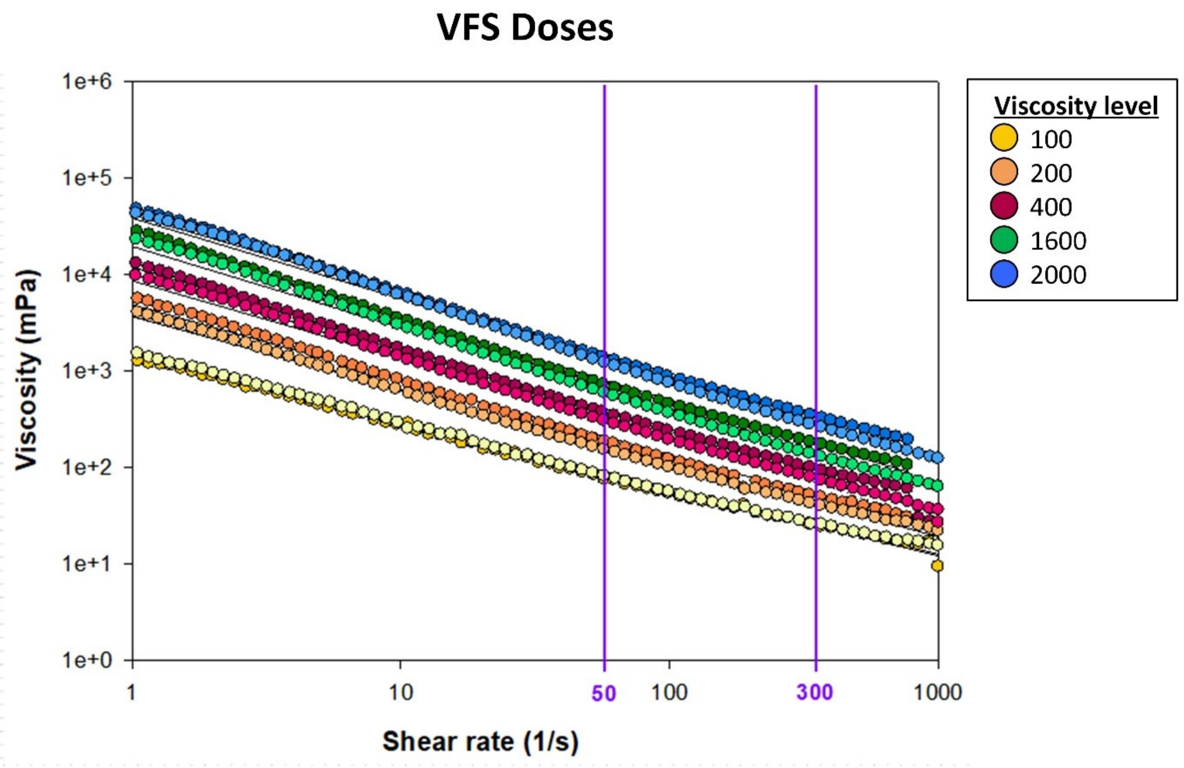

Figure S2. Viscosity curves from a shear rate range of 1–1000 s^{-1} for videofluoroscopy doses before and after oral incubation in healthy volunteers. Dark colors correspond to viscosity levels pre-oral incubation. Soft colors correspond to viscosity post-oral incubation. Purple lines mark the two main shear rate landmarks (50 and 300 s^{-1}) during deglutition in patients with oropharyngeal dysphagia.

Supplementary Information S1: Normalized protocol to determine shear viscosity for thickening

1. Material and equipment

- Solvent: thickening product
- Dissolvent: mineral water, alimentary fluid, X-ray contrast
- Equipment to measure shear viscosity: viscometer or rheometer
- Stirrer: Metallic spatula
- Container: Glass beakers or clear plastic cups
- Chronometer
- Plastic Syringe (minimal volume: 20 mm)

2. Participants

In order to perform the *ex vivo* salivary amylase effect, participants will be needed to

3. Preparation mode

a) Weigh the dissolvent in a clear plastic cup
b) Weigh the solvent in another cup
c) Initiate the chronometer and add the solvent to the dissolvent over 5 s while stirring at 4 rps with a metallic spatula
d) Continue stirring for 30 s at the same velocity

e) Leave the prepared sample resting for 10min

3. Sample analysis

In vitro shear rate effect

f) Analyze viscosity by increasing shear rate from 0 to 1000 s^{-1} in a 10 minute test at 25ºC

g) Interpolate the viscosity values at 50 and 300 s^{-1} to obtain the apparent viscosity value in the oral and pharyngeal phase, respectively

h) Repeat the process from step 'a' to 'e' two times more to obtain triplicates

Ex vivo salivary amylase effect

f) Volunteer / patient preparation: give to the patient water to clean the mouth performing intra-oral movements for 10 s. Remove the water.

g) Take 15 ml of the prepared sample with a plastic syringe and give it to the volunteer / patient. Ask them to maintain the sample in the oral cavity by performing intra-oral movements for 30s and then to spit it

h) Analyze viscosity by increasing shear rate from 0 to 1000 s^{-1} in a 10 minute test at 25ºC

i) Interpolate the viscosity values at 50 and 300 s^{-1} to obtain the apparent viscosity value in the oral and pharyngeal phase, respectively

j) Repeat the process from step 'a' to 'e' two times more to obtain triplicates

k) Repeat the process for the needed number of participants
